# Prevalence and factors associated with stunting among children aged 6–59 months in Kabale district, Uganda

**DOI:** 10.1186/s40795-022-00578-9

**Published:** 2022-08-15

**Authors:** Musa Kasajja, Elizabeth Nabiwemba, Henry Wamani, Saul Kamukama

**Affiliations:** 1 School of Public Health, Makerere University College of Health Sciences, P.O. Box 7072, Kampala, Uganda; 2 Department of Community Health and Behavioral Sciences, Makerere University School of Public Health, Kampala, Uganda

**Keywords:** Stunting, Malnutrition and under-nutrition

## Abstract

**Background:**

Despite of the global efforts undertaken to improve nutrition, malnutrition still continues to be a serious public health concern. Malnutrition in its various forms has been closely associated to major causes of illness, disability and death. Malnutrition in the form of childhood stunting has therefore been identified as a significant hindrance to human development. The aim of this study was to assess the nutritional status of children aged 6**–**59 months and determine factors associated with a high prevalence of stunting (48%) among children in Kabale district.

**Methodology:**

A cross sectional study was conducted among 640 children, aged 6**–**59 months selected using both simple random and systematic random sampling techniques. Interview administered questionnaires were used to collect household data whereas anthropometric data was collected using height boards, digital weighing scales and Mid Upper Arm Circumference (MUAC) Tapes. Nutrition status data was analyzed using ENA for SMART, 2011 and then exported to STATA version 12.0 for further analysis.

**Results:**

The overall prevalence of stunting among children 6–59 months was 41.1%. Factors independently associated with stunting included; age of the child (children in the age category of 36-47 months APOR = 0.38; 95% CI 0.18–0.79 and those in the age category of 24-35 months APOR = 0.42; 95% CI 0.19–0.88), major source of food for the household that is children from households in which mothers indicated market as the major source of food (APOR = 0.67; 95% CI 0.48–0.94) and disposal of child stool that is children whose stool was put/ rinsed in a latrine (APOR = 0.41; 95% CI: 0.23–0.74) as well as those that whose stool was thrown in garbage (APOR = 0.29; 95% CI: 0.12–0.72).

**Conclusion:**

The prevalence of stunting among children aged 6–59 months in Kabale district was high. Practices/ factors independently associated with stunting among children aged 6–59 months included; age of the child, major source of food for the household and disposal of child stool. Addressing these factors requires a proper mix of both community and health based interventions. There is also need to strengthen on strategies for reducing stunting like; sanitation and hygiene as well as food and nutrition security within rural households.

## Background

Despite of the global efforts undertaken to improve nutrition, malnutrition still continues to be a serious public health concern [1]. Malnutrition in its various forms such as; stunting, wasting, underweight and overweight has been closely associated to major causes of illness, disability and death [2] thus impeding economic advancement in most developing countries. Malnutrition in the form of childhood stunting has therefore been identified as a significant hindrance to human development [3].

Stunting has generally been described as low height-for-age (HAZ), that is a child having less than minus two standard deviations of the new World Health Organization (WHO) growth Standards [4]. Stunting reflects a process of failure to reach linear growth potential as a result of suboptimal health and/ or nutrition conditions. Stunting is now a well-established risk marker of poor child development, especially in Sub-Saharan Africa. According to WHO, the worldwide variation of the prevalence of stunting is considered to range from 5 to 65% among less-developed countries. In developing countries, the prevalence of stunting starts to rise at about three months of age and then slows down at around three years of age [5]. It has been noted that 90% of the world’s stunted children live in 36 developing countries [6] of which Sub-Saharan Africa and South Asia are home to three quarters of the world’s stunted children [7]. Despite an encouraging global downward trend in the prevalence of stunting, the progress is not the same across countries [8] especially in Sub-Saharan Africa and South Asia.

Stunting is therefore thought to result from both the nutritional experience of the individual (child) over a period of time as well as the nutritional status of the parents, particularly the mother through birth weight [9]. For instance it has been highlighted that intrauterine growth restriction due to maternal under-nutrition estimated by rates of low birth weight accounts for 20% of childhood stunting [10]. Stunting and other forms of under-nutrition reduce a child’s chance of survival, while also hindering optimal health and growth. Stunting before the age of two years predicts poorer cognitive and educational outcomes in later childhood and adolescence [11] of which these have significant educational and economic consequences at the individual, household and community levels.

In Uganda, findings show that the nutritional status of the population is generally poor especially among children less than five years, children of school going age, adolescents and women of reproductive age [12]. Data from the 2011 Uganda Demographic and Health Survey (UDHS) reports levels of child and maternal under-nutrition which have not changed much over the past 15 years. For instance 33% of children less than five years in Uganda are stunted which means that over and above 2.3 million young children are chronically malnourished, 14% are underweight and 5% are wasted. While there has been some reduction in the prevalence of child malnutrition, the change has been slow.

This study therefore assessed the prevalence and factors associated with stunting among children aged 6–59 months in Kabale district thus understanding why stunting was high in that part of Uganda.

## Methodology

### Study site, design and sample size

The study was carried out in Kabale district. The district has a population of 490,227 of which children under five years are 90,692 [13]. Two sub-counties (Ikumba and Hamurwa) out of 16 were studied reasons being that these sub-counties posted the highest number of stunted children as per the district findings from a survey that was conducted in all sub-counties in November, 2014 [14]. The study employed a cross-sectional study design and quantitative methods of data collection were used. A sample of 640 children aged 6–59 months was selected using simple random and systematic sampling procedures. The inclusion criteria included all children aged 6–59 months with their biological mothers aged 15–49 years who had lived in the study area for a period of more than five years, and consented to participate in the study as proxy respondents. Mothers with very sick children and women who were living with children that did not belong to them were excluded.

### Sampling procedure

The study used both simple random and systematic sampling. Lists of all the villages in the two sub-counties that is Ikumba (59 villages) and Hamurwa (64 villages) were generated with the help of the District Biostatistician. From a combined total of 123 villages in the two sub-counties, 32 villages were randomly selected- that is 14 villages in Ikumba and 18 villages in Hamurwa. Within each village, 20 households were selected using systematic random sampling. The first household was arrived at using the Lot Quality Technique. Subsequent households were then selected using a sampling interval of three where two households had to be skipped after the first visited household. In circumstances where the visited household did not have eligible participants we had to go to the immediate household whose front door was physically closest to the one just visited. In households that had more than one child aged 6–59 months, children were assigned numbers out of which one was randomly selected to participate in the study.

### Data collection procedure and tools

While in the community, research assistants conducted interviews using the local language (Rukiga) after obtaining written informed consent from respondents. Child’s age was obtained from birth certificates. In cases where the birth certificates were missing, age was determined through probing and using the age in months calendar. Anthropometric measurements for weight, height and MUAC were taken using Seca digital weighing scales (Model 874), Shorry height boards (Model S0114540) and adult MUAC tapes respectively. Weighing scales were tested for consistency every morning using a 2 kg stone, calibrated to zero and put on a flat surface for accurate measurements. Respondents were then asked to stand on the weighing scale without shoes and feet a little wide apart. Tethered weight was taken for children who could not stand and readings rounded off to the nearest 0.1 kg. The height board was placed either vertically or horizontally on a flat surface. Length was taken for children below 24 months in a recumbent position of which height was taken for children above 24 months and adults, respondents were asked to stand straight on a vertically placed height board while facing the individual taking the height reading. The obtained readings were rounded off to the nearest 0.1 cm. MUAC readings were obtained from both the mothers and children of which the mid-point of the arm had to first be obtained. The less active arm for each respondent had to be determined, then a reading of the upper arm’s length in centimeters from the shoulder bone to the elbow joint was determined while the arm was folded at 90 degrees. The obtained reading was then divided into two equal parts to get the mid-point, it is at that part of the upper arm that flexible tapes designed to take both adult and child MUAC were passed around dropped arms and the readings determined. MUAC measurements were also recorded to the nearest 0.1 cm.

Other variables such as socio-demographic factors and child feeding practices were obtained using an interview administered semi-structured questionnaire. On average one interview lasted for 30 min.

### Data management and statistical analysis

Raw data was cross-checked for completeness, cleaned and entered into a computer using EpiData 3.1. Nutrition data was analyzed using ENA for Smart version, 2011 after which it was categorized and exported to STATA version 12.0 for bivariate and multivariate analysis in relation to the other collected household data. Data analysis involved running frequencies and statistical associations. Prevalence odd ratios (POR) were used to test for associations. Factors with a *P* value ≤ 0.2 at bivariate analysis, and those that were biologically plausible were subjected to the backward and forward stepwise method at multivariate analysis to determine factors independently associated with stunting. Factors with a *P* value of < 0.05 were considered as statistically significant.

### Ethical considerations

Ethical approval for the study was obtained from Makerere University School of Public Health Higher Degrees Research and Ethics Committee as well as the Uganda National Council of Science and Technology (FWA 00,011,353). While in Kabale district, permission was obtained from the District Health Officer (DHO), Sub-county Chiefs and Local Council Chairpersons. Detailed information on study procedures and purpose were explained to mothers. Informed written consent was obtained from each mother before they participated in the study. Children who were found to be malnourished (severely wasted and underweight) were referred to the nearest health center of which mothers were encouraged to take their children for treatment. The study used numbers for identification of participants and the responses of the mothers were kept confidential.

## Results

A total of 640 children participated in this study, the ratio of male to female children was 1:1. The mean age of children was 26.4 months with a standard deviation (SD) of ± 14.05 and median age of 23.47 months. About half of the children who participated in the study were residing in Hamurwa sub-county 56.2% (360), the rest were from Ikumba 43.7% (280). Baseline and socio demographic characteristics of the study population are presented in Table 1 below.

**Table 1:** Characteristics of the Study Population

Variable	Number (*n* = 640)	Percentage (%)
**CHILD CHARACTERISTICS**		
**Sex of the child**		
Male	316	49.38
Female	324	50.63
**Age in months**		
6–11	106	16.56
12–23	224	35.00
24–35	151	23.59
36–47	93	14.53
48–59	66	10.31
**Child’s birth order**		
1	127	19.84
2–3	217	33.91
4–5	163	25.47
> 5	133	20.78
**Nutrition status of Children**		
Stunting (HAZ < -2 Z-score)	263	41.1
Underweight (WAZ < -2 Z-score)	49	7.7
Wasting (WHZ < -2 Z-score)	8	1.3
**RESPONDENTS CHARACTERISTICS**		
**Sex of the household head**		
Male	607	94.84
Female	33	5.16
**Mothers’ age in years**		
15–19	25	3.91
20–24	165	25.78
25–29	188	29.38
30–34	117	18.28
≥ 35	145	22.66
**Marital Status of the Mother**		
Single	11	1.72
Married	428	66.88
Cohabiting	173	27.03
Widowed	12	1.88
Divorced/ Separated	16	2.50
**Mothers’ education level**		
None	70	10.94
Primary	494	77.19
Secondary	70	10.94
Some tertiary	6	0.94
**Mothers’ Occupation**		
Farmer	622	97.19
Petty trade	18	2.81
**Major source of food for the household**		
Own production	371	57.97
Market	269	42.97
**Household size**		
2–4	254	36.69
5–7	275	42.97
> 8	111	17.34
**Area of residence (Sub-county)**		
Ikumba	280	43.75
Hamurwa	360	56.25

Prevalence of stunting (HAZ < -2 z-score) among children was 41.1%. Males had a slightly higher prevalence of stunting 42.2% compared to females 40.0%. Prevalence of underweight (WAZ < -2 z-score) among children was 7.7%. Underweight was highest among females 9.3% compared to males 6.0%. Prevalence of wasting (WHZ < -2 z-score) was 1.3% in the study population. Wasting was highest among males 9% compared to females 0.6%.

### Factors associated with stunting

Factors independently associated with stunting among children aged 6–59 months were; age of the child (children in the age category of 36–47 months; APOR = 0.38 and those in the age category of 24–35 months; APOR = 0.42), major source of food for the household that is children from households in which mothers indicated market as the major source of food (APOR = 0.67) and disposal of child stool that is children whose stool was put and or rinsed in a latrine (APOR = 0.41) as well as those that whose stool was thrown in garbage (APOR = 0.29). The results are presented in Table 2 below.

**Table 2:** Overall Factors Associated with Stunting among Children Aged 6–59 Months

Variable	Stunting		Bi-variable Analysis	Multivariable Analysis
	**Yes n (%)**	**No n (%)**	**Crude POR (95% CI)**	**Adjusted POR (95% CI)**
**Sex of the child**				
Female	130 (40.0)	195 (60.0)	1.00	
Male	133 (42.2)	182 (57.8)	0.91 (0.66- 1.23)	0.83 (0.59- 1.16)
**Age of the child**				
48–59	15 (22.7)	51 (77.3)	1.00	
36–47	44 (47.3)	49 (52.7)	**0.33 (0.16- 0.66)**	**0.38 (0.18- 0.79)**
24–35	79 (52.3)	72 (47.7)	**0.27 (0.14- 0.52)**	**0.42 (0.19- 0.88)**
12–23	104 (46.4)	120 (56.6)	0.34 (0.18- 0.64)	0.56 (0.26- 1.17)
6–11	21 (19.8)	85 (80.2)	1.19 (0.56- 2.52)	1.83 (0.74- 4.52)
**Major source of food for household**				
Own production	139 ( 37.5)	232 (62.5)	1.00	
Market	124 (46.1)	145 (53.9)	**0.70 (0.509- 0.964)**	**0.67 (0.48–0.94)**
**Person feeding the index child**				
Mother	42 (30.0)	98 (70.0)	1.00	
Father	3 (75.0)	1 (25.0)	0.14 (0.014–1.413)	0.31 (0.30- 3.13)
Elder sibling	1 (20.0)	4 (80.0)	1.71 (0.186–15.79)	3.36 (0.36- 31.8)
Child eats by him/ herself	217 (44.2)	274 (55.8)	0.54 (0.362–0.809)	0.82 (0.49- 1.35)
**Disposal of child stool**				
Child uses latrine	33 (28.4)	83 (71.6)	1.00	
Put/ rinsed in latrine	172 (43.7)	222 (56.3)	**0.51 (0 .327–0.805)**	**0.41 (0.23- 0.74)**
Buried	40 (40.8)	58 (59.2)	0.58 (0.326–1.019)	1.59 (0.32- 1.13)
Thrown in garbage	18 (56.2)	14 (43.8)	**0.31 (0.138–0.693)**	**0.29 (0.12- 0.72)**

*POR (95% CI)* Prevalence Odds Ratio and its 95% Confidence interval. *P*-values ˂ 0.05 are statistically significant

## Discussion

This study is reflective of a predominantly rural population in southwestern Uganda. The study provides important insights about stunting and factors associated with stunting among children aged 6–59 months in Kabale district. Prevalence of stunting in the study population varied according to the different age groups and decreased with an increase in age, overall prevalence was 41.1%. This prevalence was almost the same as for the entire regional average of 41.7% reported in the 2011 UDHS, high compared to findings from previous studies in Uganda [13–15] and low compared to other countries in Sub-Saharan Africa and South Asia of which these include; Malawi, Ethiopia, DR Congo, Tanzania, Nigeria, India, Pakistan, Bangladesh and Nepal [7, 16–19]. Stunting has been linked with long-term consequences such as low education attainment, increased risk of non-communicable diseases, reduced work productivity and other socio-economic disadvantages [11]. The 2013 lancet series highlight that under-nutrition is estimated to reduce a nation’s economic advance by at least eight percent through direct productivity losses, losses via poor cognition and losses via reduced schooling [20, 21].

The factors that were significantly associated with stunting included; child’s age, major source of food for the household and disposal of child stool. Findings revealed that the prevalence of stunting was slightly higher among male children (42.2%) although this was not statistically significant. It has been noted that in Sub-Saharan Africa, male children are more likely to become stunted than females of which this may be attributed to vulnerabilities in health inequalities [22].

Findings also revealed that children in the age categories of 36–47 and 24–35 months were more likely to be affected by stunting compared to children in other age groups. This is consistent with a study that was carried out in Southern Ethiopia where children more likely to be stunted included those between 24–35 months [23]. In another study carried out in Acholi sub-region, the prevalence of stunting among children aged 6–59 months peaked at 30–41 months [15]. However, in other studies, children more likely to be stunted were in the age categories of 11–23 and 12–23 [24, 25]. It should be noted that low height-for-age in children aged 24–35 months reflects a continuing process of failing to grow (stunting) whereas for older children above 36 months, low height-for-age reflects a state of having failed to grow (being stunted).

Major source of food for the household was used as a proxy for household food security. Children in households that bought their food from the market were more likely to be stunted compared to those in households that grew their own food. This indicates that households which bought food from the village markets and or nearby shops as their major source of food did not have enough food to feed their children on. A study done in Malaysia showed that children in food-insecure households were three times more likely to be stunted than children in food-secure households [26]. Another study carried out in Colombia showed that the risk for child stunting increased in a dose–response way as food insecurity became more severe [27]. This finding was expected because the study was done before the harvest season.

Children whose stool was put/ rinsed in a latrine and those whose stool was thrown in garbage were more likely to be affected by stunting compared to those that used a latrine. It has been noted that access to improved sanitation and proper disposal of child’s stool have conflicting associations with stunting, especially among the older group of children. On one hand, children from households with improved sanitation are less likely to be stunted. On the other hand, children from households where their stool is properly contained and or rinsed in a latrine are more likely to be stunted even after controlling for other variables, including sanitation [28]. This finding implies that mothers’ hygiene practices (like not washing their hands after disposing off child stool) have an effect on child health ultimately leading to stunting.

This study had a number of limitations. We did not consider seasonal variations and their effect on a range of factors affecting stunting among children in different households, though the study was conducted during a rainy season before harvest time. There might have been potential recall bias among respondents answering questions relating to events that happened in the past, especially to do with infant and young child feeding (IYCF) practices. However, questions to do with IYCF were asked based on a 24 h recall so as to minimize the effect of recall bias. Information on some potential factors or confounding variables such as parasitic infestation which is wide spread among children was not assessed reason being that this required a longer period of time and a lot of resources. This being a cross-sectional study, causality could not be established for any of the associated variables.

## Conclusion

The overall prevalence of stunting among children aged 6–59 months in Kabale district was high (41.1%). However, there was a decrease in the prevalence by 7 percentage points compared to what was reported in 2014 (48%). Practices/ factors independently associated with stunting among children aged 6–59 months were; age of the child, major source of food for the household and disposal of child stool. The risk of stunting increased with age hence the highest risk of stunting being among children aged 36–47 months. Children in households that bought food from the market and or nearby shops were more likely to be stunted. The findings also revealed that unhygienic child stool disposal (throwing of stool in garbage) was positively associated with stunting. Addressing these factors requires a proper mix of both community and health based interventions. There is also need to strengthen on strategies for reducing stunting like; sanitation, and hygiene as well as food and nutrition security within rural households.

## Data Availability

The datasets used and/ or analyzed during the current study are available from the corresponding author on reasonable request.

## Abbreviations

APOR: Adjusted Prevalence Odds Ratios
CI: Confidence Intervals
DHO: District Health Officer
HAZ: Height for Age Z score
IYCF: Infant and Young Child Feeding
MUAC: Mid Upper Arm Circumference
POR: Prevalence Odds Ratios
UDHS: Uganda Demographic and Health Survey
WAZ: Weight for Age Z score
WHO: World Health Organization
WHZ: Weight for Height Z scores
